# Primary or metastatic branchial cleft carcinoma?: a case report

**DOI:** 10.3389/fsurg.2023.1205287

**Published:** 2023-09-20

**Authors:** Yang Ma, Hangyu Liu, Lijing Yang, Shijie Tang, Lungang Shi

**Affiliations:** ^1^ Department of Plastic Surgery, Meizhou Clinical Institute of Shantou University Medical College,Meizhou, China; ^2^ Department of Plastic Surgery and Burn Center, the Second Affiliated Hospital of Shantou University Medical College, Shantou, China

**Keywords:** branchial cleft carcinoma, primary branchial cleft carcinoma, metastatic branchial cleft carcinoma, head and neck tumor, head and neck (H&N) cancer

## Abstract

The brachial cleft carcinoma is an extremely rare head and neck facial malignancy, and there is some disagreement about its differential diagnosis. In this paper, we report a 63-year-old male patient who had a mass on the left side of the neck and diagnosed as the brachial cleft carcinoma by intraoperative biopsy pathology. However, this patient was diagnosed with the carcinoma of the left soft palate more than 20 days after surgery and esophageal cancer 2 years later, and was treated accordingly. Therefore, it is hard to confirm whether the branchial cleft carcinoma is primary or metastatic. In fact, the diagnostic criteria for primary squamous cell carcinoma of branchial cleft cysts are very rigorous. Confirmation of the diagnosis is based on pathological examination of the branchial cleft cyst epithelium lined with squamous cells, meanwhile, a thorough examination should also be performed to exclude the presence of other primary cancers.

## Introduction

Branchial cleft carcinoma originates as a carcinoma of the epithelium lining the branchial cleft cyst, which is extremely low in incidence and difficult to diagnose accurately. The majority of its pathological type is squamous epithelial cell carcinoma. Branchial cleft cysts are congenital malformations caused by malformation of the first to fourth branchial cleft. Depending on the degree of malformation during embryonic development, these abnormalities may appear as fistula, cyst, or sinus tract (1). The most common type of branchial cleft cyst comes from the second cleft, accounting for approximately 40%–90% of the four branchial cyst types (2),while the first, third and fourth cleft have much rarer abnormality. The typical branchial cleft cyst is located on the anterior borderline of the sternocleidomastoid muscle. Although the branchial cleft carcinoma is uncommon, it remains a difficult challenge to carefully differentiate the primary branchial cleft carcinoma and those that metastasize to them. The preferred treatment is radical resection with adjuvant radiotherapy or chemotherapy (3, 4).

## Case presentation

**Figure 1:**
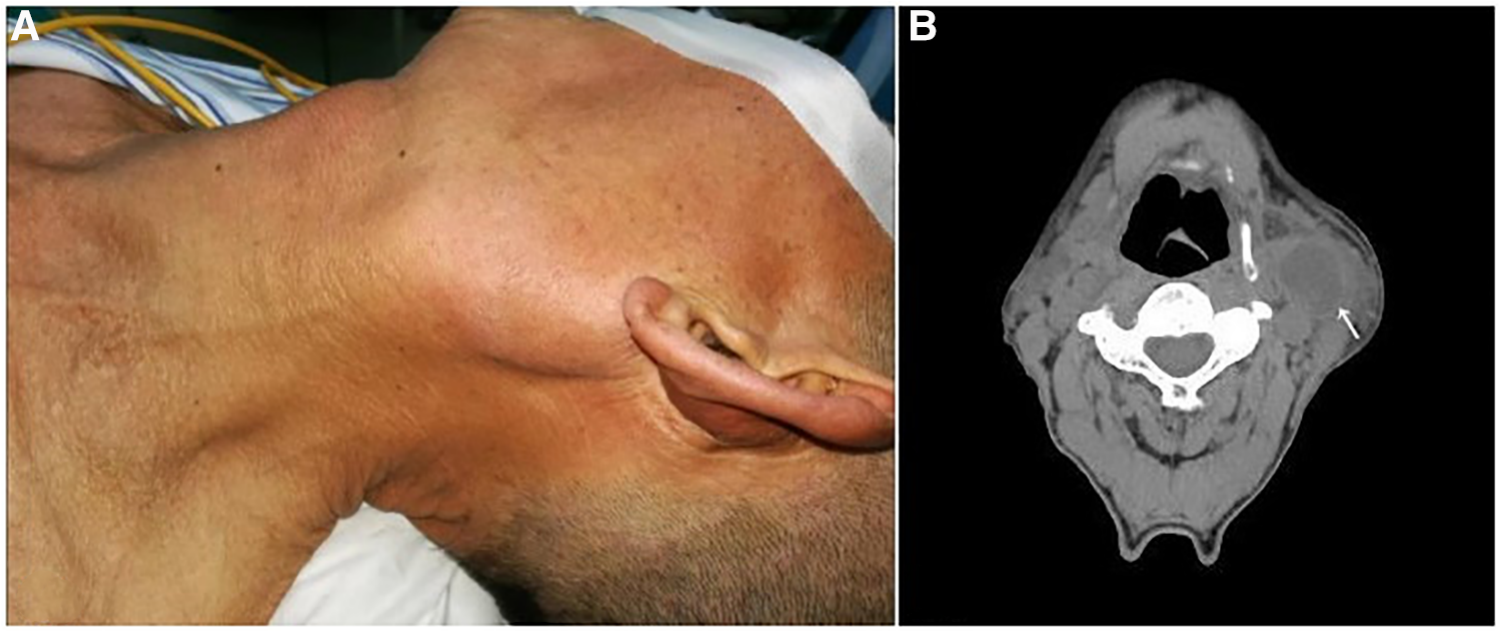
(**A**) The general view before resection. (**B**) The CT image of the branchial cleft cyst.

**Figure 2:**
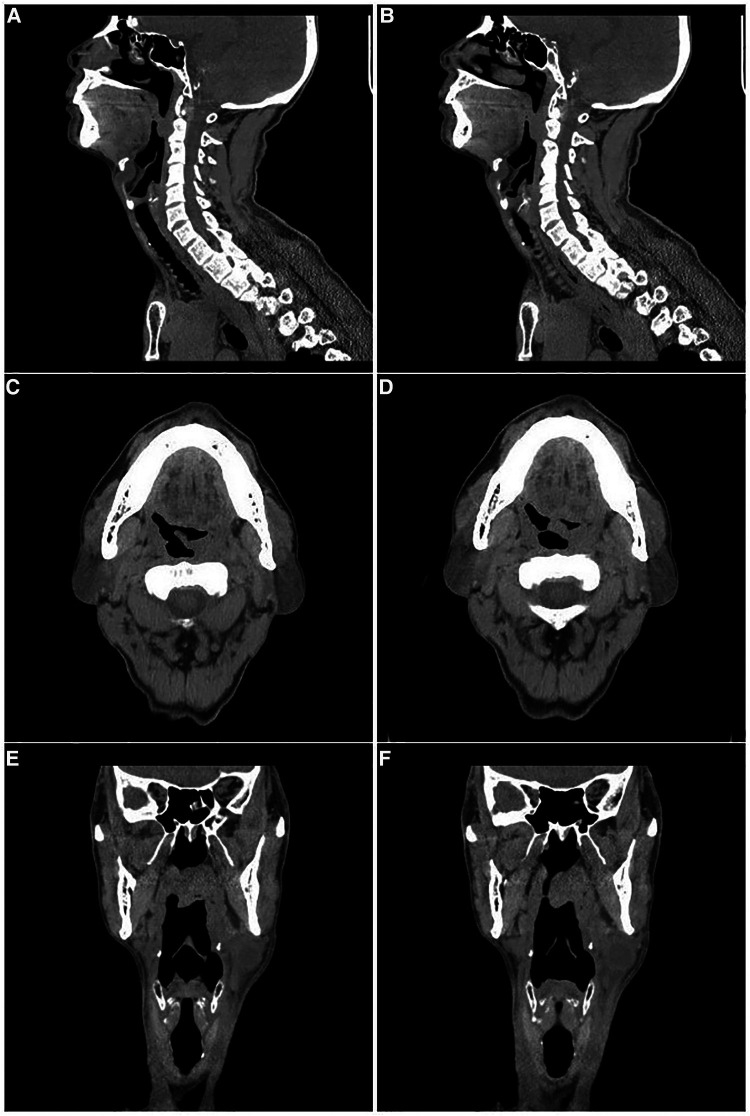
The CT images of the left soft palate. (**A**)–(**B**): Sagittal view. (**C**)–(**D**): Transverse view. (**E**)–(**F**): Coronal view.

A 63-year-old male patient was admitted to our department on December 25, 2017, with a “left neck mass found for 3 years.” The mass was approximately 6 cm in diameter with clear boundaries, protruding from the skin, hard and poorly mobile (Figure 1A), and no enlarged lymph nodes were palpable on the clavicle area behind the ear. Old pulmonary tuberculosis of the left upper lung was found, with no history of surgery, trauma, or drug allergy. According to the CT report, there was a 26 × 18 × 37 mm mass occupying the left upper neck below the parotid gland (Figure 1B), which was considered branchial cleft cyst with infection. In addition, the mucosa of the left piriform fossa was thickened, which was considered to be an inflammatory lesion (Figure 2). Surgical treatment of a left neck mass was performed on December 26, 2017, under intravenous complex general anesthesia with tracheal intubation.

**Figure 3:**
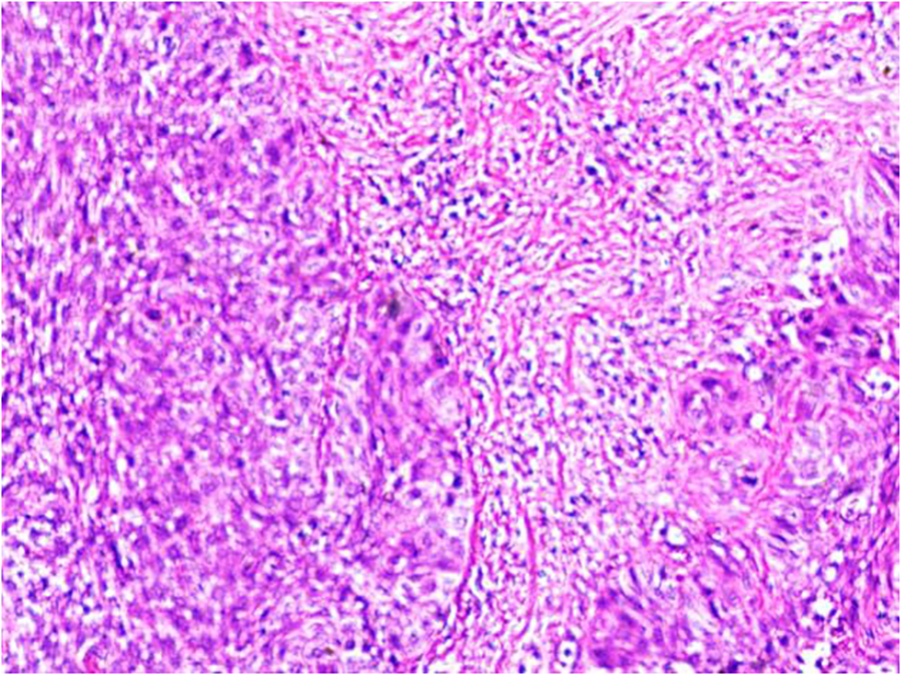
Microscopic view of cystic structures.

Then pathological results were confirmed by the pathology department as a carcinoma of the left cervical branchial cleft cyst, which was a squamous cell carcinoma (branchial cleft carcinoma). Microscopically, cystic structures were seen, lined with squamous epithelium, and lymphocytic bands were seen around the epithelium with lymphoid follicle formation. The lined epithelium was locally heterogeneous, breaking through the basement membrane to infiltrate the surrounding area, and was distributed in nested clusters (Figure 3). The cells were of different sizes, with thickened nuclear chromatin, some cells with obvious nucleoli, and some keratinized beads were visible. The patient was admitted to Cancer Hospital for further treatment.

**Figure 4:**
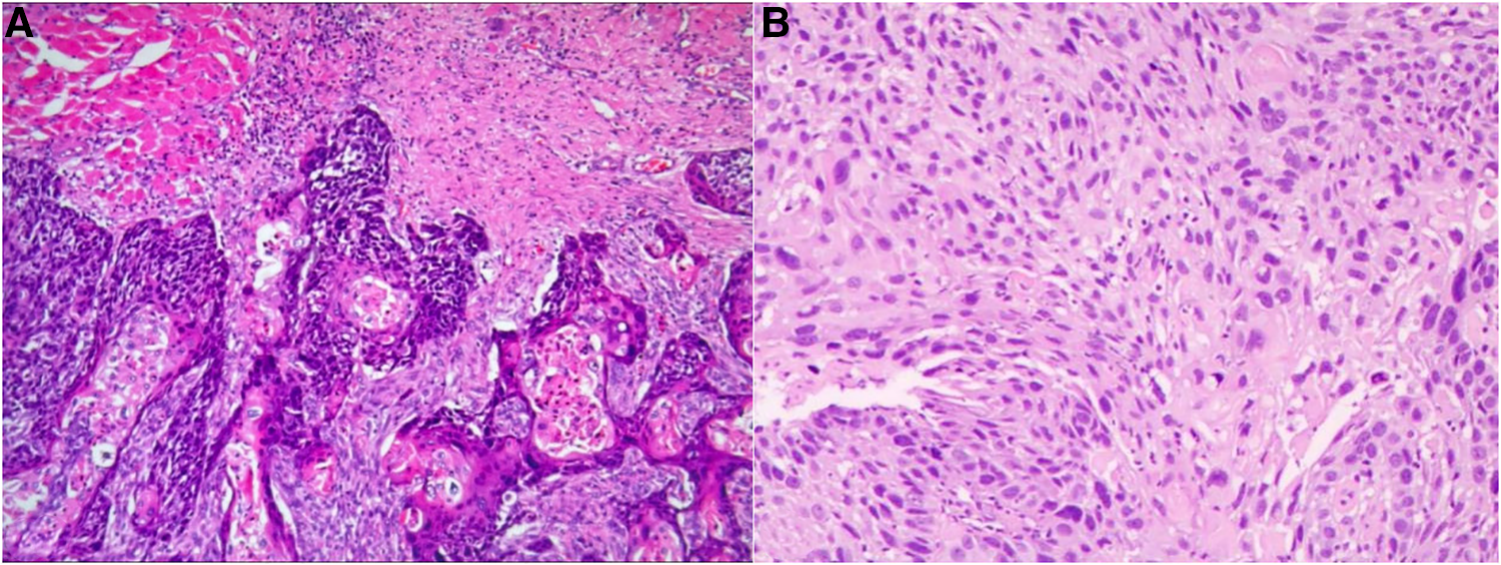
(**A**) Microscopic view of the left soft palate. (**B**) Microscopic view of esophageal tissue.

Twenty days after the first surgery, the patient was diagnosed with the squamous carcinoma of the left soft palate at Cancer Hospital after a tissue biopsy and immunohistochemical examination. Radical excision of left soft palate carcinoma was performed on January 17, 2018, under general anesthesia with pneumonectomy. Pathological examination microscopically revealed highly differentiated squamous cell carcinoma of the left soft palate infiltrating the transverse muscle (Figure 4A). Postoperative radiotherapy was performed after the operation.

On April 7, 2020, the patient was readmitted to our gastroenterology department with “obstruction of swallowing for more than 10 days.” He presented as vomiting after eating, and the vomit was the food eaten. CT in other hospitals showed dilatation of the upper esophagus with irregular wall thickening and luminal narrowing, considering the possibility of esophageal mass occupancy. Then esophageal cancer was diagnosed by pathological examination. A biopsy was taken from the esophagus 20 cm from the incisor, and the pathological diagnosis was squamous cell carcinoma. Microscopically, heterogeneous cells were seen under the squamous epithelium in the form of nesting sheets, with varying cell sizes, increased nucleoplasm ratio, thickened chromatin, eosinophilic nuclei and nuclear fission images, and localized keratinized bead formation (Figure 4B).

## Discussion

The origin of branchial cleft cysts has been debated for a long time, but it is generally considered to be formed by incomplete degeneration of fetal branchial organs during embryonic development. Volkman first reported branchial cleft carcinoma in 1882. The primary branchial cleft carcinoma is extremely rare, and its histopathological diagnosis is based on the transformation of normal epithelium within the branchial cleft cyst to malignant epithelium lined with squamous epithelium (5, 6). Some researchers believe that branchial cleft carcinoma is actually a cystic metastasis of oral cancer rather than a carcinoma originating from the branchial cleft (7, 8). In 1950, Martin (4) and other researchers proposed diagnostic criteria for the cystic carcinoma of the gill slit: (i) the tumor should occur on the line extending from the front of the tragus down to the anterior border of the sternocleidomastoid muscle; (ii) the histology should be consistent with the origin of the residual tissue of the branchial cyst; (iii) the patient must survive at least 5 years through regular examination and must not develop other primary tumors; (iv) the squamous carcinoma is visible within the epithelium of the cyst. However, some scholars (9) still consider the criterion of absence of other primary tumors during the 5-year follow-up too idealistic and of limited diagnostic and therapeutic value.

In this case, the patient was diagnosed with squamous cell carcinoma of the left soft palate more than 20 days after resection for squamous cell carcinoma of the left branchial cleft cyst, and then diagnosed with the esophageal carcinoma 2 years later. It is unclear whether there is an association between the carcinoma of the left soft palate and esophagus and the previous branchial cleft of the left neck. In terms of the diagnostic criteria described above, this case is not diagnostic as primary branchial cleft carcinoma. In addition, according to the pathological results, the patient's esophageal cancer was more likely to be metastatic cancer. It has been shown that in cases of lymph node metastasis of squamous cell carcinoma, approximately 72%–90% of the primary tumors were found to be located in Waldeyer's ring, which is the base of the tongue, palatine tonsils and nasopharyngeal region (5, 10, 11). Resection of the neck mass and histopathological examination is the only accurate basis for the diagnosis of true malignancy, although its origin may remain unknown.

Branchial cleft carcinoma is rare and difficult to diagnose and differentiate, therefore, the current knowledge of this disease is mainly based on case reports. Since the current treatment for this disease is mainly radical surgery with post-operative radiotherapy or chemotherapy to prevent tumor recurrence (12). More relevant clinical studies and histopathological data would be beneficial to deepen the understanding of the branchial cleft carcinoma, which has an important role in improving the guidance of the treatment for this disease.

## Conclusion

The confirmation of primary squamous cell carcinoma of branchial cleft cysts is very rigorous, which is based on pathological examination of the branchial cleft cyst epithelium lined with squamous cells. Meanwhile, a thorough examination should also be performed to exclude the presence of other primary cancers. This case report also suggests that it is necessary to pay attention to the physical examination of the oral cavity when diagnosing a neck mass.

## Data Availability

The original contributions presented in the study are included in the article/Supplementary Material, further inquiries can be directed to the corresponding author.
